# Authentication of *Marantodes pumilum* (Blume) Kuntze: A Systematic Review

**DOI:** 10.3389/fphar.2022.855384

**Published:** 2022-06-08

**Authors:** Ida Syazrina Ibrahim, Mazlina Mohd Said, Noraida Mohammad Zainoor, Jamia Azdina Jamal

**Affiliations:** ^1^ Drug and Herbal Research Centre, Faculty of Pharmacy, Universiti Kebangsaan Malaysia, Kuala Lumpur, Malaysia; ^2^ National Pharmaceutical Regulatory Agency, Petaling Jaya, Malaysia

**Keywords:** authentication, identification, fingerprinting, *Marantodes pumilum*, quality control

## Abstract

Botanical drug products consist of complex phytochemical constituents that vary based on various factors that substantially produce different pharmacological activities and possible side effects. *Marantodes pumilum* (Blume) Kuntze (Primulaceae) is one of the most popular Malay traditional botanical drugs and widely recognized for its medicinal use. Many studies have been conducted focusing on the identification of bioactive substances, pharmacological and toxicological activities in its specific varieties but less comprehensive study on *M. pumilum* authentication. Lack of quality control (QC) measurement assessment may cause different quality issues on *M. pumilum* containing products like adulteration by pharmaceutical substances, substitution, contamination, misidentification with toxic plant species, which may be detrimental to consumers’ health and safety. This systematic literature review aims to provide an overview of the current scenario on the quality control of botanical drug products as determined by pharmacopoeia requirements specifically for *M. pumilum* authentication or identification. A systematic search for peer-reviewed publications to document literature search for *M. pumilum* authentication was performed using four electronic databases: Web of Science, PubMed, Scopus and ScienceDirect for related studies from January 2010 to December 2021. The research studies published in English and related articles for identification or authentication of *M. pumilum* were the main inclusion criteria in this review. A total 122 articles were identified, whereby 33 articles met the inclusion criteria. Macroscopy, microscopy, chemical fingerprinting techniques using chromatography, spectroscopy and hyphenated techniques, and genetic-based fingerprinting using DNA barcoding method have been used to identify *M. pumilum* and to distinguish between different varieties and plant parts. The study concluded that a combination of approaches is necessary for authenticating botanical drug substances and products containing *M. pumilum* to assure the quality, safety, and efficacy of marketed botanical drug products, particularly those with therapeutic claims.

## Introduction

Since ancient times, botanical drugs have been employed in the daily lives of the world population due to their medicinal efficacy in promoting well-being and health. Around 80% of the world’s population consumes botanical drugs as health supplements since they are thought to be effective in disease management and have been recognized as safe for decades owing to their natural origin. Despite the widespread use of botanical drug products for a long time, problems with quality control persist. The increased demand for botanical drug products may expose them to various types of adulteration, such as substitution, contamination, or the use of fillers, all of which represent a threat to the health and safety of consumers (Techen et al., 2014; Abubakar et al., 2018). In fact, different countries define botanical drug products differently and use different systems for registering, licensing, dispensing, manufacturing, and trading them to ensure their safety, efficacy, and quality. As a result, there is a disparity in registration requirements between countries and variation in the botanical drugs quality.

Recognizing the need, for the past few decades, WHO has consistently issued various guidelines and policies related to botanical drugs, such as Guidelines for the Assessment of Herbal Medicines, Good Agricultural and Collection Practices (GACP) and Quality Control Methods for Medicinal Plant Materials, with the goal of standardizing and harmonizing botanical drugs regulation globally (Yadav and Dixit, 2008). Currently, botanical drug products must meet pharmacopoeia identification requirements for organoleptic evaluation (touch, smell, sight, and taste), macroscopic evaluation (shape, color, and texture), microscopic assessment and chemical fingerprint techniques such as chromatography and spectroscopy (Li et al., 2020). Additionally, the British Pharmacopoeia has included DNA barcoding as a means of identifying botanical drugs (Heinrich and Anagnostou, 2017). In fact, Malaysia’s regulatory body has consistently adopted various international guidelines issued by the World Health Organization (WHO), Medicines and Healthcare Products Regulatory Agency (MHRA), European Medicines Agency (EMA), Therapeutic Goods Administration (TGA), and Food and Drug Administration (FDA) to strengthen the registration requirements for botanical drug products since 1992. It is vital to identify and authenticate botanical drugs and products utilizing a variety of approaches, whether during the final product phase for clinical study evaluation or throughout product development for the market (Smillie and Khan, 2010).

**FIGURE 1:**
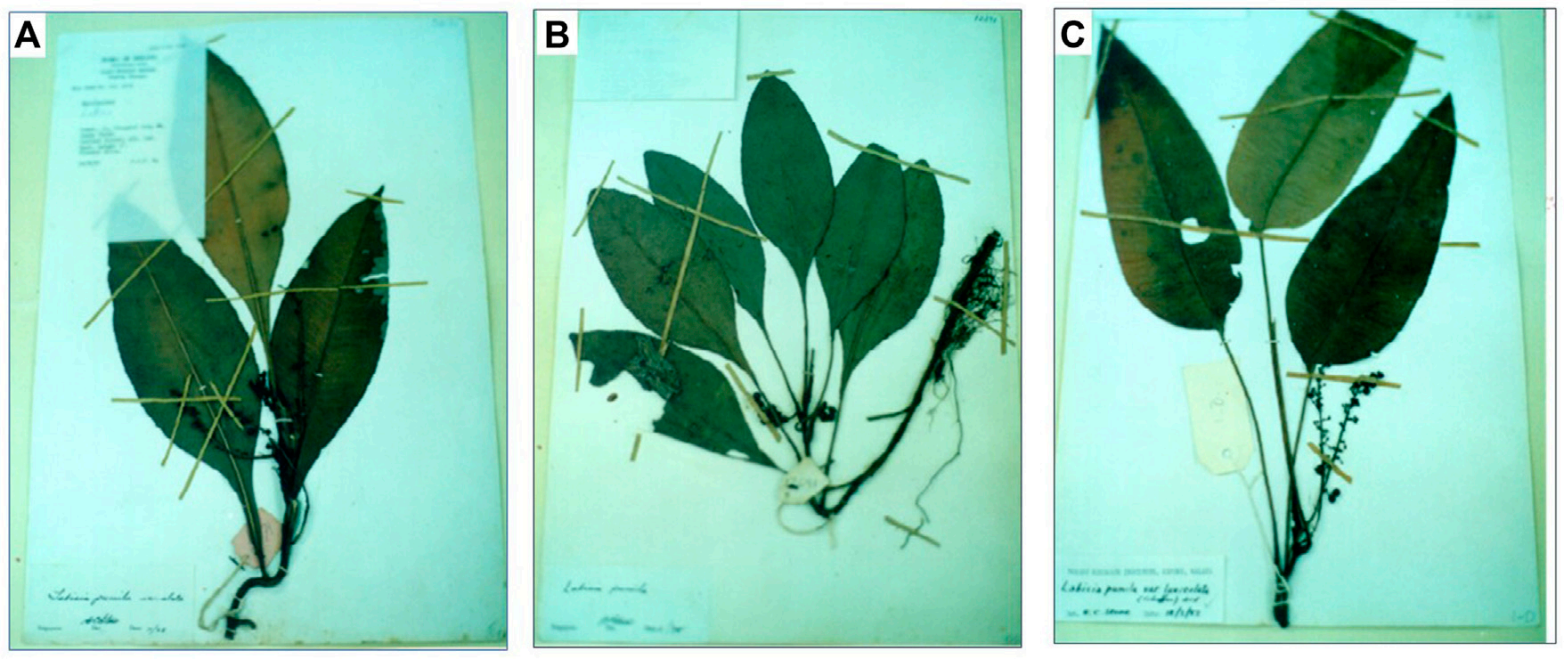
Voucher specimens of **(A)** *Marantodes pumilum* var. *alata*, **(B)** *Marantodes pumilum* var. *pumila* and **(C)** *Marantodes pumilum* var. *lanceolata* (obtained from the Kepong Herbarium of Forest Research Institute Malaysia).

Due to the open online market, the botanical drug-based industry has also piqued the interest of Asian countries. Malaysia’s forest is home to a diverse array of medicinal plants with a great potential for use in the botanical drugs industry. Malaysia’s botanical drugs domestic market was expected to grow at a 15% annual rate from RM7 billion in 2010 to around RM29 billion by 2020 (Fadzil et al., 2018). The increase in the number of botanical drug products registered with the National Pharmaceutical Regulatory Agency (NPRA) demonstrates the growing demand for botanical drug products (Fadzil et al., 2018). Realizing the huge economic opportunities in the local botanical drugs industry and the requirements that need to be complied with, the agricultural National Key Economic Areas (NKEA) Entry Point Project 1 (EPP1) was focused on potential growth that might contribute to Malaysia’s gross national income (GNI). Due to their potential therapeutic properties, Malaysia’s government has identified 11 important plants, including *Eurycoma longifolia* Jack (Simaroubaceae), *Marantodes pumilum* (Blume) Kuntze (Primulaceae), *Andrographis paniculata* (Burm.f.) Nees (Acanthaceae), and others, to be commercialized as high-value botanical drug products. *M. pumilum*, locally known as Kacip Fatimah, is widely spread in Southeast Asian tropical forests and is well-known for its medicinal properties. It is a member of the Primulaceae family and was formally recognized as a member of the family Myrsinaceae and known as *Labisia pumila* (Blume) Fern.-Vill (WFO, 2022). In various parts of Malaysia, *M. pumilum* is referred to as kachip patimah, selusuh fatimah, rumput siti fatimah, akar fatimah, kachit fatimah, pokok pinggang, rumput palis, tadah matahari, mata pelandok rimba, bunga belangkas hutan (Chua et al., 2011) and sangkoh (Iban) (Abdullah et al., 2013). There are eight *M. pumilum* varieties and only three varieties; var. *alata* (Scheff.) Mez., var. *pumila* and var. *lanceolata* (Scheff.) Mez. are widely distributed in Malaysia rain forest and have attracted the researcher’s interest thus far (Sunarno, 2005; Chua et al., 2012). The three varieties can be distinguished by their petioles and leaf characteristics. *M. pumilum* var. *alata* has red veins and broad winged petioles, whereas var. *pumila* has an emarginate winged petiole and an ovate leaf blade, and var. *lanceolata* has a long, non-winged or terete petiole (Figure 1). However, due to the close macromorphological features, it was extremely difficult to visually separate them based on petiole characteristics, particularly when the petioles were not fully formed (Abdullah et al., 2012).

Traditionally, indigenous women of the Malay Archipelago consumed water decoctions of *M. pumilum* to aid in labor and delivery, while the botanical drug is believed to tone the abdominal muscles, assist in tightening the birth canal, and enhance overall body strength during postpartum (Zakaria and Mohd, 1994). Additionally, ancient communities employed the *M. pumilum* to treat diarrhoea, rheumatism, gonorrhea and flatulence (Burkill, 1966). Numerous research has shown that *M. pumilum* has a wide range of pharmacological activities, including antibacterial, antifungal, anti-inflammatory, cytotoxicity, antioxidative, xanthine oxidase inhibitory, phytoestrogenic, anticarcinogenic, anti-aging, anti-hyperuricemia, anti-osteoporotic, anti-obesity, cardioprotective effect, and uterotonic (Norhaiza et al., 2009; Choi et al., 2010; Karimi et al., 2011, 2013; Pihie et al., 2011; Jamal et al., 2012; Mamat et al., 2014; Pandey et al., 2014; Dianita et al., 2016; Hairi et al., 2018; Wan Omar et al., 2019; Aladdin et al., 2020; Rahmi et al., 2020).

Phytochemical constituents found in *M. pumilum* varieties include flavonoids, phenolics, methyl gallate, carotenoids, ascorbic acids, fatty acids, saponins, alkenyl compounds and benzoquinone derivatives (Ahmad et al., 2018). Many variables influence the phytochemical constituents, such as environmental factors, species varieties and plant parts. The variation in phytochemical contents between batches often results in markedly variable pharmacological actions and probable side effects (Liang et al., 2004; Goodarzi et al., 2013). According to Karimi et al. (2011), the phytochemical constituent presence and abundance differs between *M. pumilum* varieties and plant parts. The study indicated that gallic acid was highest in var. *alata* leaves, followed by var. *lanceolata* leaves and var. *pumila* leaves. Several studies have established that different *M. pumilum* species and plant parts possess distinct pharmacological properties, including phytoestrogenic activity of var. *alata* leaves (Giaze et al., 2018; Hairi et al., 2018), xanthine oxidase inhibitory activity of var. *pumila* leaves (Aladdin et al., 2020) and anti-inflammatory effect of var. *pumila* roots (Rahmi et al., 2020).

Due to the wide range of phytochemicals found in this plant that can contribute to different pharmacological effects and side effects, majority of research on *M. pumilum* has focused on the bioactive substances, pharmacological and toxicological activities and less studies conducted specifically on its identification and authentication. As such, the aim of this review is to present an overview of the current state of authentication for *M. pumilum* in the global botanical drug products industry.

## Materials and Methods

### Search Strategy

A systematic review of the literature was conducted in accordance with the Preferred Reporting Items for Systematic Reviews and Meta-Analyses (PRISMA) guideline (Moher et al., 2009). A search strategy based on a combination of relevant keywords and Boolean operators was used [ALL = (authentication or identification or quality control or chemical profiling or fingerprint) and (marantodes pumilum or labisia pumila or kacip fatimah)] for Web of Science database and [(“authentication” OR “identification” OR “quality control” OR “chemical profiling” OR “fingerprint”) AND (“marantodes pumilum” OR “labisia pumila” OR “kacip Fatimah”)] for PubMed, Scopus and ScienceDirect databases. Following the search conducted on 18 November 2021, the option “search alert” was selected to receive weekly updates for all four literature databases. All the selected articles were saved in Mendeley Desktop Version 1.19.8 (2008–2020) reference manager.

### Selection Process and Criteria

*Identification*: Database searches identified 606 records (WoS = 22, PubMed = 4, Scopus = 499 and ScienceDirect = 81).

*Screening*: Articles were screened manually in four stages. Initially, articles that were published as a review, book chapter, or conference proceeding were excluded. Secondly, articles that were published between January 2010 and December 2021 were considered. Thirdly, articles without data or information on the identification or authentication of *Marantodes pumilum* (Kacip Fatimah) as botanical drug substances or botanical drug products were omitted. Following screening, 552 articles were deleted. Finally, duplicate entries were removed from the databases, leaving 37 eligible articles.

*Eligibility*: A total of 37 full-text papers were evaluated and screened for eligibility using the following criteria:

1. The tested sample was required to be made up of botanical drug substances or botanical drug products. A variety of scientific names, that is, *Labisia pumila* (synonym), *Marantodes pumilum*, and the common name Kacip Fatimah were accepted for the review.
2. The details of the tested sample, including the collection site, plant species, plant parts and sample processing, were clearly documented and described.
3. All pertinent methodologies within the scope of the studies were accepted. The tested samples were authenticated using several techniques, including macroscopic and microscopic methods, chemical fingerprinting, genetic fingerprinting, and phytochemical analysis.

**FIGURE 2:**
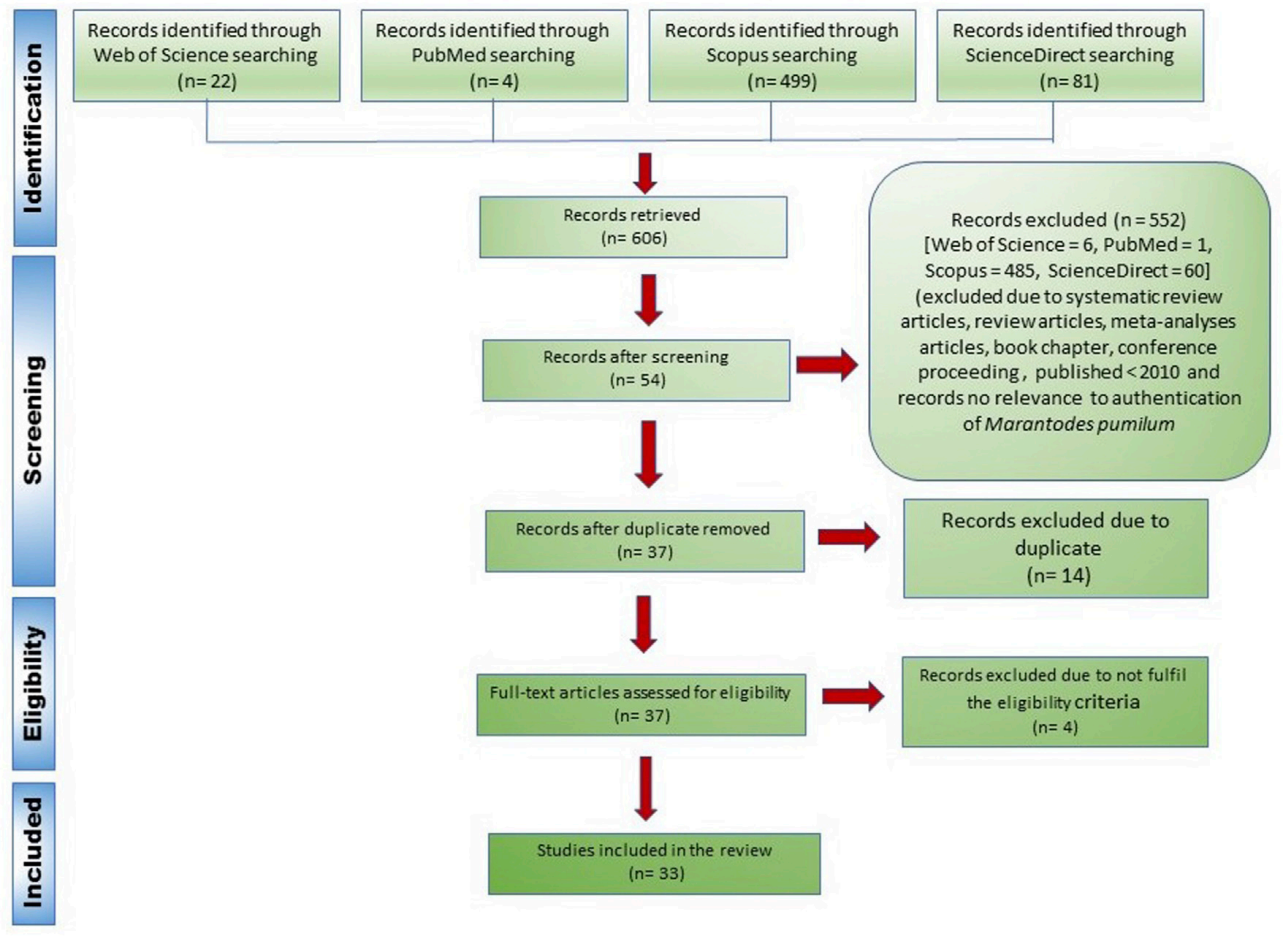
Flowchart of the article search process according to the PRISMA guidelines (Moher et al., 2009).

*Included:* 33 peer-reviewed articles were included in the systematic review of the literature, as described in Figure 2. The flowchart was made in accordance with PRISMA guidelines (Moher et al., 2009).

## Results

All selected articles were published between January 2010 and December 2021 (Table 1). Thirty-three full-text articles met the inclusion criteria and were classified as research articles in this review. Majority of the included full-text articles were conducted locally at various universities and institutions in Malaysia, while six were conducted abroad in China (study 24), Sweden (studies 1 and 5), the United States of America (studies 6 and 7) and India (study 2). Even though the genus *Labisia* was reclassified as *Marantodes* in 2012, 81% (*n* = 27) of publications cited *Labisia pumila* rather than *Marantodes pumilum*. Four studies were conducted on all three common varieties of *M. pumilum* var. *alata*, var. *pumila*, and var. *lanceolata*; one study used *M. pumilum* var. *alata* and var. *pumila*, sixteen studies focused exclusively on *M. pumilum* var. *alata*, one study used only *M. pumilum* var. *pumila* and ten studies made no mention of the *M. pumilum* variety. On average, 70% of publications indicated the variety used in the study, with the remaining 30% of articles lacking identification at variety level. Apart from that, 60.6% (*n* = 20) studies used *M. pumilum* leaves, whereas other studies used whole plants (21.2%, *n* = 7), leaves and stem/roots (15.15%, *n* = 5), and stem-roots (3%, *n* = 1). According to the review, (72.7%, *n* = 24) majority of the researchers used wild plant sources rather than cultivated sources.

**TABLE 1:** Authentication and identification methods of *Marantodes pumilum* botanical drug substances and commercial products.

No.	Study	Species and sources	Voucher specimen	Identification/Authentication Methods
1	Mannerås et al. (2010)	Whole plant of *L. pumila* var. *alata,* (Wild plant from rainforest of Peninsular Malaysia)	Not available	Not available
2	Pandey et al. (2010)	Leaf of *L. pumila,* (Wild plant from Perak)	21648	Morphology
3	Pattiram et al. (2011)	Leaf of *L. pumila,* [Wild plant (unkown source)]	Not available	GCMS
4	Chua et al. (2011)	Leaf of *L. pumila* var. *alata, L. pumila* var. *pumila,* [Cultivated plant from Forest Research Institute Malaysia (FRIM)]	FRI 59810	Phytochemical screening, UPLC/ESI/MS/MS, chemometrics
5	Fazliana et al. (2011)	Whole plant of *L. pumila* var. *alata*, (Wild plant from Pahang)	FRI54816	Morphology
6	Ali and Khan (2011)	Root of *L. pumila,* (Cultivated plant supplied by Holista Biotech Sdn. Bhd.)	PID26	Morphology, NMR, IR, TLC, HPLC
7	Avula et al. (2011)	Leaf and stem-root of *L. pumila* var. *alata,* (Cultivated plant supplied by Holista Biotech Sdn. Bhd.)	3809, 3810, 8400, 5002, 5003, 5004 (United States )	TLC, IR, HPLC/UV/ELSD, LC/ESI/TOF, NMR, HRESI/MS
8	Al-Mekhlafi et al. (2012)	Leaf of *L. pumila,* (Wild plant from Pahang)	ACP0084/08	UV, IR, NMR
9	Abdullah et al. (2012)	Leaf of *L. pumila* var. *alata, L. pumila* var. *pumila* and *L. pumila* var. *lanceolata,* (Wild plant from Negeri Sembilan, Johor, Pahang, and Kedah)	Not available	IR, chemometric analysis
10	Pan et al. (2012)	Whole plant of *L. pumila,* (Wild plant from Perak)	I/LP/3547	Morphology, HPLC
11	Abdah et al. (2014)	Leaf of *L. pumila* var*. alata,* (Cultivated plant from FRIM)	Not Available	HPLC
12	Effendy et al. (2014)	Whole plant of *L. pumila* var*. alata*, (Wild plant from Kedah)	Not available	Morphology, phytochemical screening
13	Tnah et al. (2014)	Leaf of *L. pumila* var*. alata,* var*. pumila* and var. *lanceolata*, (Wild plant from Pasoh Forest Reserve, Negeri Sembilan)	KEP 223663—223665	Morphology, DNA barcoding
14	Norhayati et al. (2014)	Whole plant of *L. pumila* var*. alata,* [Wild plant (unknown source)]	Not available	HPLC
15	Karimi et al. (2015)	Leaf of *L. pumila* var*. alata, L. pumila* var*. pumila* and *L. pumila* var*. lanceolata*, (Wild plant from Sungkai, Perak; Hulu Langat, Selangor; and Kota Tinggi, Johor)	Not available	Phytochemical screening
16	Aladdin et al. (2016)	*M. pumilum* var. *alata, M. pumilum* var. *pumila* and *M. pumilum* var. *lanceolata,* (Wild plant from Bujang Melaka Forest Reserve, Kampar, Perak)	UKMB 30006/SM 2622, UKMB 30007/SM s.n., UKMB 30008/SM s.n, respectively	Morphology, microscopy, HPTLC, HPLC, ATR-FTIR
17	Dianita et al. (2016)	Whole plant of *L. pumila* var*. alata,* (Wild plant from Perak]	UKMB 30010	Morphology, HPLC
18	Karimi et al. (2016)	Leaf of *L. pumila* var*. alata,* [Cultivated plant from Universiti Putra Malaysia (UPM)]	Stone 6030 (KLU) (UPM)	Morphology, HPLC, GC and GC/MS
19	Adam et al. (2017)	Leaf of *M. pumilum,* (Wild plant from Perak)	KLU49047 (UM)	Morphology, LC/MS
20	Latiff et al. (2018)	Leaf of *L. pumila* var*. alata,* (Cultivated plant from FRIM)	FRI 59810	Morphology, LC/MS/MS
21	Giribabu et al. (2018)	Leaf of *M. pumilum,* (Wild plant from Tapah, Perak)	KLU 46767	Morphology, UPLC/MS/MS
22	Tnah et al. (2019)	Leaf of *L. pumila*, (Wild plant from forest reserves, botanical gardens and medicinal plant nurseries in Peninsular Malaysia and commercial products)	Not available	Morphology, DNA barcoding
23	Giaze et al. (2019)	Leaf and stem-root of *M. pumilum* var. *alata*, (Wild plant from Simpang Empat, Kedah)	UKM-HF131	Morphology, LC/MS
24	Wu et al. (2019)	Leaf and stem of *L. pumila,* (Cultivated plant from FRIM)	Not available	UPLC/MS/MS
25	Tan et al. (2019)	Leaf of *M. pumilum,* (Wild plant from Tapah, Perak)	KLU49047 (UM)	Morphology, LC/MS/MS
26	Muhamad et al. (2019)	Leaf of *L. pumila* var*. alata,* (Wild plant from Tapah, Perak)	FF/UiTM/KF/02/13	Morphology, LC/MS
27	Wan Omar et al., (2019)	Leaf of *M. pumilum* var. *alata,* (Wild plant from Tapah, Perak)	KLU49047 (UM)	Morphology, LC/MS
28	Dharmani et al. (2019)	Leaf and stem-root of *L. pumila* var*. alata,* (Wild plant supplied by Delima Jelita Herbs, Kedah)	UKMHF131	Morphology, LC/MS/MS
29	Salehan et al. (2020)	Leaf of *L. pumila* var*. alata*, (Wild plant from Alor Setar, Kedah)	Not available	LC/MS Q-TOF
30	Radzali et al. (2020)	Leaf of *L. pumila* var*. pumila,* ((unknown) from Batu Pahat, Johor)	UKMB 30007/SM sn	Morphology, HPLC
31	Manshor et al. (2020)	Aerial part and leaf of *L. pumila,* (Wild plant from Sungai Siput Utara, Perak)	11632	Morphology, HPLC
32	Yeop et al. (2021)	Whole plant of *L. pumila* var. *alata*, (Wild plant from Bentong, Pahang)	PIIUM 0321	Morphology, UPLC/PDA, UPLC/QTOF/MS
33	Tarmizi et al. (2021)	*Labisia pumila* var. *alata* and *L. pumila* var*. pumila (red and green leaf)* (Cultivated plant from Batu Pahat, Johor)	PID 250817-17 (LPPG), PID 260817-17 (LPPR), PID 270817-17 (LPA)	Morphology, HPLC, DNA barcoding

Notably, all investigations included in this review employed at least one approach for identifying or authenticating *M. pumilum*. Generally, about (66.70%, *n* = 22) of the study conducted morphological tests on the *M. pumilum* samples and 67.7% (*n* = 23) of the 33 study included the voucher specimen number for the test samples except for studies 1, 3, 9, 11, 12, 14, 15, 22, 24, and 29. Multiple sources of *M. pumilum* from different areas in Malaysia were used in 18.2% (*n* = 6) of research conducted abroad. Botanists authenticated most test samples based on morphological identification, except for those used in studies 1 and 24 that were conducted in China and Sweden, respectively, due to the lack of information on morphological identification and voucher specimen for *M. pumilum* in the articles compared to other studies. Qualified botanists in public educational institutions and government research institutions performed the plant authentication.

**FIGURE 3:**
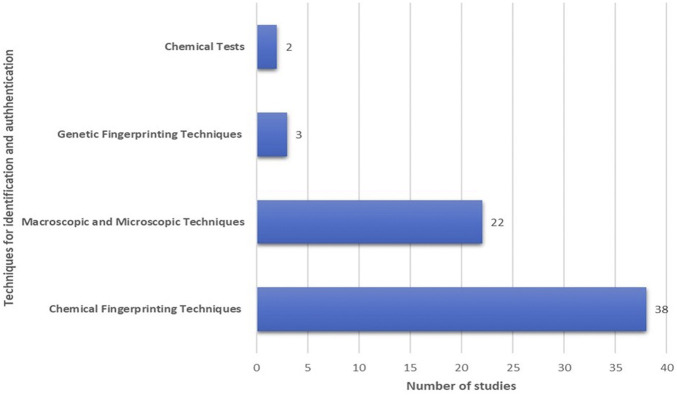
*Marantodes pumilum* identification and authentication techniques reported between 2010 and 2021.

The researchers’ choice of approaches for detecting phytochemical characteristics varies according to the sensitivity of the procedures. Most studies employed chemical fingerprinting techniques (58.46%, *n* = 38), followed by macroscopic and microscopic techniques (33.85%, *n* = 22), genetic fingerprinting techniques (4.6%, *n* = 3) and chemical tests (3.07%, *n* = 2) (Figure 3). Less sensitive techniques such as ultraviolet (UV) spectroscopy, thin layer chromatography (TLC), high performance thin layer chromatography (HPTLC) and Fourier transform infrared (FTIR) spectroscopy were complemented by high-end instruments such as liquid chromatography-mass spectrometry (LC/MS/MS) or nuclear magnetic resonance (NMR). Among the chromatographic techniques, HPLC is the mostly used method (30.30%, *n* = 10), followed by HPTLC/TLC (12.12%, *n* = 4) and gas chromatography (GC) (3.03%, *n* = 1). Over the past 10 years, a total of 36.6% (*n* = 12) studies have applied the hyphenated chromatographic and mass spectrometric techniques in their research.

Two studies (12 and 15) used phytochemical screening, whereas study 4 combined phytochemical analysis with a more advanced instrument, ultra-performance liquid chromatography coupled with electrospray ionization tandem mass spectrometry (UPLC/ESI/MS/MS), to identify the nine flavanols and nine phenolics in various fractions of *M. pumilum*. Additionally, the study 4 used a chemometric approach such as principal component analysis (PCA) to demonstrate the similarities and differences in phytochemical profiles of different fractions. Chemometrics was also used in conjunction with the chemical fingerprinting technique in study 9, which reported on the use of macroscopy, IR spectroscopy, and chemometric analysis as a powerful technique for differentiating 84 test samples from seven different locations in Peninsular Malaysia. The first method for simultaneous determination of triterpenes, saponins and alkenated-phenolics in the leaves, stems, and roots of *M. pumilum* var. *alata* was developed in study 7 using high performance liquid chromatography-ultraviolet-evaporative light scattering detector (HPLC-UV-ELSD) in conjunction with other structure elucidation techniques such as TLC, IR, liquid chromatography coupled with electrospray ionization quadrupole time of flight mass spectrometry (LC/ESI/TOF), NMR and high resolution electrospray ionization mass spectrometry (HRESI/MS). Spectroscopic and chemical analyses were used in study 6 to elucidate the structures of alkyl phenols and saponins found in the roots of *M. pumilum*. Three investigations (studies 13, 22. and 33) used DNA fingerprinting to identify *M. pumilum*.

Table 2 summarises selected characteristic features derived from the 33 articles used to distinguish *M. pumilum* varities using various analytical techniques.

**TABLE 2:** Characteristic features to distinguish *Marantodes pumilum* varieties based on different analytical techniques.

No.	Techniques	Plant Parts	Analysis		*M. pumilum* var. *alata*	*M. pumilum* var. *pumila*	*M. pumilum* var. *lanceolata*	References
1	Microscopic analysis (anatomy)	Leaf epidermis	Type of trichome	Adaxial epidermis	Absent	Simple, 2-armed, scale	Absent	Aladdin et al. (2016)
				Abaxial epidermis	Scale, capitate glandular	Scale	Scale	
			Type of stomata	Adaxial epidermis	Absent	Absent	Absent	
				Abaxial epidermis	Anisocyatic, staurocytic	Anisocyatic, diacytic	Anisocyatic	
			Pattern of anticlinal walls	Adaxial epidermis	Straight to curved	Straight to curved	Straight to curved	
				Abaxial epidermis	Straight to wavy	Straight to wavy	Straight to wavy	
		Leaf venation	Marginal venation		Incomplete	Incomplete	Marginal venation	
			Areolar venation		Closed, a few opened, minority free ending veinlets	Closed, free ending veinlets	Areolar venation	
		Leaf lamina and margin	Main mascular bundle		Equidistant to abaxial and adaxial epidermis	Equidistant to abaxial and adaxial epidermis	Close to the adaxial epidermis	
			Marginal outline		Rounded	Rounded	Tapering	
			Marginal direction		10–30° upwards	10–30° upwards	30–45° downwards	
		Midrib	Outline	Adaxial	Slightly convex	Flat/straight	Flat/straight	
				Abaxial	U-shaped	¾ of circle	¾ of circle	
			Cell inclusion		Solitary crystal calcium oxalate (rectangular), druses, scattered starch grains	Solitary crystal calcium oxalate (cubic), clustered starch grains	Solitary crystal calcium oxalate (cubic), druses, scattered starch grains	
			Type of trichome		Scale, capitate glandular	Scale, capitate glandular	Scale	
		Petiole	Outline		Wing presence at the left and right of adaxial side, ¾ of oval at abaxial side	Wing presence at the left and right of adaxial side, ¾ of oval at abaxial side	Oval	
			Cell inclusion		Brachyscelereids, solitary crystals calcium oxalate (cubic), druses	Brachyscelereids, starch grains, solitary crystals calcium oxalate (cubic), druses	Starch grains, solitary crystals calcium oxalate (cubic), druses	
		Stem	Type of trichome		Scale	Scale, capitate glandular	Scale	
			Outline		Circular	Circular	Circular	
			Parenchyma cortex		Ca. 10–20	Ca. 8–10	Ca. 8–10	
			Number of additional vascular bundle in cortex		6	6	5	
			Pith		Relatively wide	Relatively medium	Relatively medium	
			Cell inclusion		Brachyscelereids, solitary crystals, druses, starch grains	Brachyscelereids, solitary crystals, druses, starch grains	Brachyscelereids, solitary crystals, druses, starch grains	
			Type of trichome		Scale	Scale, capitate glandular	Scale, capitate glandular	
			Secretory canals		Present in pith parenchyma	Present in pith and parenchyma cortex	Present in pith and parenchyma cortex	
2	FTIR with KBR disk	Leaf	IR spectra		1733 (C = O stretching)	1733 (C = O stretching)	Absent	Abdullah et al. (2012)
					1204 (C-H in plane deformation)			
			Second derivative IR spectra	1597 (C = C stretching)	Present	Absent	Absent	
				1331 (O-H bending)	Absent	Absent	Present	
			2D correlation IR spectra	1660 (C = O vibration)	Present	Absent	Present	
				1559 and 1600	More intense	Low intensity	Low intensity	
				Cross-peaks at (1600, 1640), (1560, 1640) and (1560, 1600)	Strong intensity	Low intensity	Low intensity	
				Cross-peaks at (545–688, 662–740)	Strong broad	Weak	Narrow
			Principal component analysis (PCA)	The varieties are clustered differently			
3	ATR-FTIR	Leaf	IR spectra		3341 (O-H stretching)	3340 (O-H stretching)	3285 (O-H stretching)	Aladdin et al. (2016)
					1242 (C-O stretching)	1235 (C-O stretching)	1237 (C-O stretching)	
						1157 (C-O stretching)	1159 (C-O stretching)	
		Stem-root			3326 (O-H stretching)	3329 (O-H stretching)	3330 (O-H stretching)	
					1614 (C-C stretching)	1615 (C-C stretching)	1611 (C-C stretching)	
					1021 (C-O stretching)	1021 (C-O stretching)	1021 (C-O stretching)
4	HPTLC	Leaf	Fingerprint chromatogram		Presence of peaks at R_f_ 0.20–0.70			Aladdin et al. (2016)
		Stem-root			Presence of peaks at R_f_ 0.22 at different intensities			
5	HPLC	Whole plant	Detection of phytochemical compounds on chromatogram		• Ardisicrenoside B	No information	No information	Avula et al. (2011)
					• Ardisiacrispin A			
					• 3-O-α-l-rhamnopyranosyl-(1 → 2)-β-d-glucopyranosyl-(1 → 4)-αl-arabinopynanosyl cyclamiretin A			
					• Ardisimamilloside H			
					• Irisresorcinol			
					• Belamcandol B			
					• Demethylbelamcan-daquinone B			
		Leaf			• Gallic acid	• Gallic acid	No information	Tarmizi et al. (2021)
					• Rutin	• Rutin		
					No information	• Gallic acid	No information	Radzali et al. (2020)
						• Methyl gallate		
						• Caffeic acid		
					• Belamcandol B, 5-pentadec-10′-(Z)-enyl resorcinol	No information	No information	Muhamad et al. (2019)
					• 1,3-dihydroxy-5- pentadecylbenzene, 5-(heptadec-12′-(Z)-enyl) resorcinol			
					• Demethylbelamcanda-quinone B			
					• Quercetin	No information	No information	Latiff et al. (2018)
					• Myricetin			
					• Gallic acid	No information	No information	Karimi et al. (2016)
					• Pyrogallol			
					• Myricetin			
					• Quercetin			
					• Naringin			
					• Daidzein			
					• Catechin			
					• Epicatechin			
					• Gallic acid	No information	No information	Salehan et al. (2020)
					• Gallic acid	No information	No information	Abdah et al. (2014)
6	LCMS	Whole plant	Detection of phytochemical compounds on chromatogram		• Gallic acid	No information	No information	Giaze et al. (2019)
					• Caffeic acid			
					• Ellagic acid			
					• Apigenin			
					• Kaempferol			
					• Quercetin			
					• Myricetin			
		Whole plant			• Ardisicrenoside B	No information	No information	Avula et al. (2011)
					• Ardisiacrispin A, 3-O-α-l-rhamnopyranosyl-(1 → 2)-β-d-glucopyranosyl-(1 → 4)-αl-arabinopynanosyl cyclamiretin A			
					• Ardisimamilloside H			
					• Belamcandol B			
					• Demethylbelamcanda-quinone B			
					• Irisresorcinol			
		Leaf			• Benzoic acid	No information	No information	Chua et al. (2011)
					• Gallic acid			
					• Vanillic acid			
					• Syringic acid			
					• Salicylic acid			
					• Cinnamic acids			
					• Protocatechuic acid			
					• Coumaric acid			
					• Caffeic acid			
					• Chlorogenic acid			
					• Quercetin			
					• Myricetin			
					• Kaempferol			
					• Catechin			
					• Epigallocatechin			
7	DNA Genetic Fingerprinting	Leaf	DNA References barcode		*ITS*2 (MK249864) *rbc*L (MH828448)	*ITS*2 (MK249864) *rbc*L (MH838008)	*ITS*2 (MH749147) *rbc*L (MH766971)	Tarmizi et al. (2021)
					*rbcL*			Tnah et al. (2019)
					*trnH-psbA*			
			Microsatellites		84 alleles	48 alleles	66 alleles	Tnah et al. (2014)

## Discussion

Between 2016 and 2020, almost 3,000 botanical drug products were registered, increasing by 16% and accounting for 50% of all registered products in Malaysia within 5 years (NPRA, 2020). The figures indicate the significant growth of botanical drug products in the Malaysian market because of persistent client demand. Authentication of botanical drugs is critical to ensuring the consistency of their quality, safety, and efficacy prior to production. Numerous experts have emphasized the importance of certification of botanical drugs to assure their purity and safety. As a result, rigorous authentication must be performed to ascertain the quality and safety of botanical drug products prior to registration approval. Standardization and quality control of raw materials must follow the pharmacopoeia-defined process of identification and authentication.

The systematic review assessed current quality control trends for *M. pumilum* in the botanical drug research and development setting, as described by pharmacopoeia. In general, the 33 peer-reviewed articles demonstrate that a variety of techniques have been used to identify and authenticate different varieties and plant parts of *M. pumilum*. Most of the articles reviewed collected wild *M. pumilum* specimens. The continued reliance on raw materials derived from wild resources, whether for research or commercial purposes, will eventually deplete the supply of *M. pumilum*. This has become a source of concern for botanical drugs suppliers, as wild *M. pumilum* is known to grow slowly in its natural habitat. As a result, several research institutions have conducted extensive tissue culture breeding for *M. pumilum* to keep up with the expanding market demand (Tarmizi et al., 2021).

Additionally, it is discovered that most of the reviewed studies employed at least one method of identification, and that awareness of the requirement has gradually increased since 2010. Effective identification and authentication tools are critical for monitoring the source of high-demand raw materials to avoid undesirable activities such as adulteration of raw materials, which negatively impacts the quality of botanical drug products. As recently reported, this approach was widely used for ginseng products, supplements, commercial botanical drug products, and Kadsura crude drugs (Liu et al., 2019; Ichim et al., 2020; Ichim and de Boer, 2021).

Organoleptic evaluation, macroscopy, and microscopy are the first three fundamental principal methods of identification and authentication used to ensure the quality of botanical drugs (World Health Organization, 2011). Due to the morphological and phytochemical complexity of interspecific hybrids, within-species variation, and the difficulty associated with recognizing species in some plant genera, voucher specimens are critical for organoleptic and macroscopic verification of source material utilized in botanical drugs research (Eisenman et al., 2012). From this review, ten of the articles did not provide information on voucher specimens of *M. pumilum* used in the studies. Lack of adequate voucher specimens has resulted in major issues, such as the inability to duplicate crucial experimental results and the incorrect assignment of phytochemical and pharmacological data to the correct genus and species. Authentication is especially important for *M. pumilum* since previous studies have shown that different varieties, plant parts and types of extracts have varying phytochemical compositions and pharmacological actions (Karimi et al., 2015; Aladdin et al., 2020; Rahmi et al., 2020). Thus, prior to producing botanical drug products containing *M. pumilum* for a particular intended pharmacological action, accurate plant variety and plant part identification must be performed. Adhering to the pharmacopoeia monograph specification for identification tests of botanical drugs would confirm the material’s authenticity. Microscopic examination enables primary species identification, as well as the detection of adulteration, contamination, and substitution of botanical drugs (Upton et al., 2020) (Table 3). Aladdin et al. (2016) reported the first effective distinction of three *M. pumilum* varieties and plant parts utilizing microscopic approach. The microscopical characteristics, such as stomata, trichomes, stem and leaf margin, petiole, midrib, vascular system, anticlinal walls, secretory canals, and cell inclusion, can all be used to differentiate and identify each variety of *M. pumilum* and its plant part (Table 2). As previously reported, 41% of 508 botanical drug products sold in 13 countries that were microscopically authenticated were found to be adulterated (Ichim et al., 2020). However, macroscopic, and microscopic analyses alone are insufficient for reliably identifying plant species and determining their quality (Smillie & Khan 2010; Srirama et al., 2017). Plant tissues with little or no cellular variations, processed materials, and extensive dehydration of plants can eliminate diagnostic features, making analysis challenging with these methods (Smillie and Khan, 2010). The issues are compounded by the lack of appropriate reference material and a scarcity of qualified taxonomists (Ichim et al., 2020). Therefore, additional identification techniques should be included to verify the authenticity of plant materials.

**TABLE 3:** Strengths and limitations of analytical techniques for *Marantodes pumilum* authentication.

Techniques		Strengths	Limitations
Organoleptic, macroscopy and microscopy		• Quick physical evaluation for adulteration, contamination, and substitution (Upton et al., 2020)	• Conventional method, imprecise and inconsistent result (Smillie & Khan, 2010) • Requires expertise and skilled or well-trained personnel (Smillie & Khan, 2010) • Microscopy is not applicable for materials in extracted or prepared (e.g., resins) form (Upton et al., 2020)
Chemical fingerprinting	IR/NIR Spectroscopy	• Quick, non-destructive, and high throughput method with minimal sample preparation (Upton et al., 2020)	• Affected by variables such as moisture, particle size and homogeneity of test samples (Upton et al., 2020)
		• Able to generate chemical fingerprint to differentiate plant varieties and plant parts (Aladdin et al., 2016)	
	TLC	• Manual, rapid, simple, flexible, low-cost, and minimal sample preparation (Liang et al., 2004)	• Issues with reproducibility, resolutions, sensitivity and difficulty to detect trace phytochemical components (Yongyu et al., 2011)
		• Test sample and References standard can be analysed simultaneously (Liang et al., 2004)	
	HPTLC	• Automated sample application allows for improved separation, band resolution and reproducibility of results (Aladdin et al., 2016)	
		• High throughput and screen multiple samples in a single assay (Upton et al., 2020)	
	HPLC	• High selectivity, sensitivity, resolution, and fully automatable operation (Smillie and Khan, 2010)	• Unable to distinguish between closely related species or non-target species that have similar chemical profiles (Upton et al., 2020)
		• Enable qualitative and quantitative analysis (Upton et al., 2020)	• Affected by factors related to variation in climate, phenotype, storage condition, age, and cultivation time (Mohammed et al., 2017)
	MS hyphenated techniques	• Powerful for rapid identification of phytochemical constituents in plant extracts (Liang et al., 2004)	• High cost
		• High resolution, high speed, accurate mass-measurement and able to retrieve more information in a complex botanical drug substance (Yongyu et al., 2011)	
Biological fingerprinting	DNA Barcode	• Rapid, sensitive, and effective tool for identification of species (Tarmizi et al., 2021)	• Unable to identify extracted form or processed botanical drugs (Tarmizi et al., 2021)
		• Widely used to differentiate individual plant, genus, homogeneity analysis, and detection of adulterants (Upton et al., 2020)	• Highly dependent on the availability of References standard data sequences (Tarmizi et al., 2021)
		• Less affected by plant age, physiological conditions, environmental factors, harvest, storage, and processing methods (Yongyu et al., 2011)	• Unable to provide information related to concentration of compounds with therapeutic value (Yongyu et al., 2011)
			• Genome information only as a complement tool of other quality control techniques (Yongyu et al., 2011)

Numerous papers describe the use of fingerprint profiling for botanical drugs identification and authentication by spectroscopy and chromatography. Guo (2017) demonstrated that evaluating the botanical drugs quality based on a single or specified markers overlooks the synergistic effects of the multi-phytochemical components of botanical drugs, suggesting that a holistic quality assessment approach using chemical fingerprinting method is relevant. In a prior work, fingerprints of different plant parts of *Panax notoginseng* (Burkill) F.H. Chen (Araliaceae) were generated using a combination of near infrared spectroscopy (NIR), HPLC, UPLC, and capillary electrophoresis (CE) (Zhu et al., 2014). Aladdin et al. (2016) have reported the usage of a more simple, convenient, and non-destructive phytochemical fingerprinting technique, namely attenuated total reflectance-FTIR (ATR-FTIR) without the usage of KBr (Table 2). The work was an advance on a prior study that used multi-step infrared spectroscopy and a KBR disc (Abdullah et al., 2012). Even though the method without KBr has a lower resolution than the method with KBr disc (Zou et al., 2005), the results of the different profiles between the three *M. pumilum* varieties and plant parts clearly demonstrated that the ATR-FTIR fingerprinting technique can be used to determine the identity and quality control of *M. pumilum* raw materials. However, this technique alone may be best suited for a single authentic plant ingredient because it may be difficult to gain accurate information of the phytochemical compositions and to detect adulterants based only on the chemical functional groups from the infrared spectrum. Moisture, particle size, and homogeneity of test samples all have an impact on analytical precision (Upton et al., 2020) (Table 3).

The phytochemical fingerprint profile of botanical drugs generates a large amount of data in the form of chromatograms or spectra, making it nearly impossible for the analyst to visually inspect each data point and exploit the useful chemical information contained in the fingerprint data *via* univariate analysis. As a result, a multivariate data analysis technique was developed to analyze chemical fingerprinting data to eliminate or reduce undesired sources of variation caused by various variables or instrumental responses from the analytical techniques, as well as to extract useful and meaningful information from the fingerprint data (Gad et al., 2013; Huang et al., 2016). Chemometric techniques have been widely used in quality control of botanical drug products due to their capacity to tackle a variety of problems in a variety of domains, including similarity analysis and exploratory learning. Apart from that, the chemometric approach can analyze a variety of data, both qualitatively and quantitatively, *via* a classification algorithm and a multivariate calibration algorithm (Li et al., 2020). The combination of chemometric techniques such as principal component analysis (PCA) and multilayer perceptron classifier (MLPC) modelling with ATR-FTIR has been reported as a rapid and effective method for botanical drugs quality evaluation of *Gastrodia elata* Blume (Orchidaceae) powder (Zhan et al., 2022). From this systematic review study, Abdullah et al. (2012) successfully characterized leaves of three *M. pumilum* varieties using IR and chemometrics (Table 2), whereas Chua et al. (2011) used an integrated approach of chemical fingerprinting with UPLC-ESI-MS/MS and chemometric technique to differentiate *M. pumilum* var. *alata* and var. *pumila* leaves based on the compositions of nine flavonols, two flavanols, and nine phenols.

TLC technique has been widely recognized as a preliminary screening approach to HPLC, owing to its ability to rapidly generate a fingerprint of varied plant materials in a single, simple, and low-cost analysis while producing high sample throughput. TLC is used for preliminary screening or identification of phytochemical components that provide the plant’s unique fingerprint (Liang et al., 2004). The HPTLC technique, when combined with automated sample application and densitometric scanning, offers numerous advantages, including high throughput samples, low analytical costs, and the ability to separate test samples and reference standards concurrently (Upton et al., 2020) (Table 3). These methods are described in most international pharmacopoeias, including the British Pharmacopoeia, the United States Pharmacopeia, and the Chinese Pharmacopoeia. There are numerous studies that used TLC and HPTLC for authentication such as ginseng, Radix Puerariae and *M. pumilum* (Xie et al., 2006; Siyumbwa, 2015). Aladdin et al. (2016) reported that the HPTLC fingerprint profiles of three varieties of *M. pumilum* leaves and stem-roots gathered from a wild source at a single location were somewhat comparable with varying intensities, implying the presence of identical phytochemicals in varying concentrations (Table 2). According to Karimi et al. (2015), total phenolics, total flavonoids, and fatty acid concentration also varied between *M. pumilum* var. *alata*, var. *pumila*, and var. *lanceolata* leaves obtained from three separate locations from the wild. The presence of identical compounds with varying compositions complicates accurate authentication of commercial plant materials, as phytochemical compound concentrations are influenced by a variety of extrinsic factors, such as geographic (latitude, altitude, and soil type), climatic (light, temperature, rainfall, and atmospheric compositions) and agricultural practice (cultivation, harvesting and processing methods, and storage conditions), as well as intrinsic factors, such as plant age, genetics, chemotypes and botanical parts (Liang et al., 2004; Yongyu et al., 2011). *M. pumilum* has been reported to contain potential pharmacologically active phytochemicals such as quercetin, myricetin, kaempferol, naringin, rutin, apigenin, catechin, epigallocatechin, pyrogallol, gallic acid, ascorbic acid, salicylic acid, syringic acid, vanillic acid, protocatechuic acid, coumaric acid, caffeic acid, chlorogenic acid, daidzein, genistein, β-carotene, anthocyanins, demethylbelamcandaquinone B, and 3,7-dihydroxy-5-methoxy-4,8-dimethyl-isocoumarin (Norhaiza et al., 2009; Ali and Khan, 2011; Chua et al., 2011; Ehsan et al., 2011; Hairi et al., 2018; Wu et al., 2018; Aladdin et al., 2020). Gallic acid and caffeic acid were identified as the major phenolic acids in the methanol extracts of three *M. pumilum* varieties (Karimi et al., 2011). Due to their widespread presence in other plants, the two compounds cannot be considered a unique biomarker for *M. pumilum*. Hence, the diverse class of phytochemicals necessitates the use of more sensitive approaches for *M. pumilum* identification and authentication.

Liquid chromatography is one of the most efficient analytical techniques for phytochemical profiling since the stationary phase column, mobile phase gradient system and detector can all be modified to suit the analysis of a variety of phytochemical components. For the development of a validated analytical method, statistically significant representative set of plant samples from multiple populations is used to establish a fingerprint profile, whereas reference standards, whether commercially available, extracted, or isolated, are necessary (Smillie and Khan, 2010). In this review, Avula et al. (2011) used HPLC to assess the phytochemicals content isolated from *M. pumilum* var. *alata* leaves, stems, and roots (Table 2), whereas Aladdin et al. (2016) distinguished three varieties of *M. pumilum* and their plant parts based on fingerprinting profile. However, both studies utilized only botanical drugs obtained from a single location, indicating that additional research is necessary. Closely related plant species with similar chemical profiles will not be discriminated using HPLC (Table 3). Researchers have used hyphenated chromatographic and mass spectrometric techniques, such as liquid chromatography-mass spectrometry (LC/MS), gas chromatography-mass spectrometry (GC/MS), and capillary electrophoresis-mass spectrometry (CE/MS), to authenticate plant materials. In this systematic review study, LC/MS (Giaze et al., 2019), LC/MS/MS (Dharmani et al., 2019), UPLC/MS/MS (Wu et al., 2019), and LC/ESI/TOF (Avula et al., 2011) were used to determine phytochemical compositions of different *M. pumilum* plant parts, whereas UPLC-ESI/MS/MS was used to detect components between *M. pumilum* var. *alata* and var. *pumila* leaves (Chua et al., 2011). To date, the combination of chromatography and mass spectrometry is the most advanced approach and is often used by researchers to analyze the composition of botanical drugs qualitatively and quantitatively to determine the consistency of their quality. Mass spectrometry imaging may be used to visually assess quality variations (Wei et al., 2020). However, the cost of the hyphenated equipment is a significant constraint (Table 3).

The researchers are currently interested in the other tool for botanical drugs authentication using genetic fingerprinting techniques. DNA barcoding enabled a rapid examination of the botanical drugs composition and was found to be an effective technique for authenticating dried and powdered plant materials for quality control purposes (Gesto-Borroto et al., 2021). Tnah et al. (2014) were the first to report the use of a genetic-based fingerprint technique to differentiate leaves of three *M. pumilum* varieties collected in the wild, followed by another investigation of botanical drug products containing *M. pumilum* (Tnah et al., 2019) (Table 2). According to the latter study, DNA barcoding was able to detect 56.7% of 30 selected botanical drug products containing *M. pumilum* and were found to be authentic, 10% were substituted with other plant taxa, and 6.7% were contaminated. DNA information was not detected in 26.6% of botanical drug products due to the low concentration or degradation of the processed botanical drug preparations. The study reported the authenticity of a product containing a single ingredient of *M. pumilum*, however, for a product containing a mixture of *M. pumilum* and *Querqus lusitanica* Lam. (Fagaceae) had no DNA sequence. Additionally, a recent study by Tarmizi et al. (2021) effectively used DNA barcoding and HPLC techniques for the identification of cultivated *M. pumilum* var. *alata* and var. *pumila* leaves, as well as investigation of the authenticity of botanical drug products containing *M. pumilum*. However, nine of test samples (60%) reported not amplifiable due to the lack or low concentration of DNA recovered from degradation. This demonstrated that the primary limitation of the current DNA barcoding approach is its inability to identify *M. pumilum* at the variety level due to inaccuracies in selecting the targeted sequence, use of a large barcode size and DNA degradation in processed materials (Table 3). As a result, DNA barcoding may not be suitable as a stand-alone method of identification of processed botanical drug products and should be combined with additional authentication techniques such as morphological features and chemical analyses.

**FIGURE 4:**
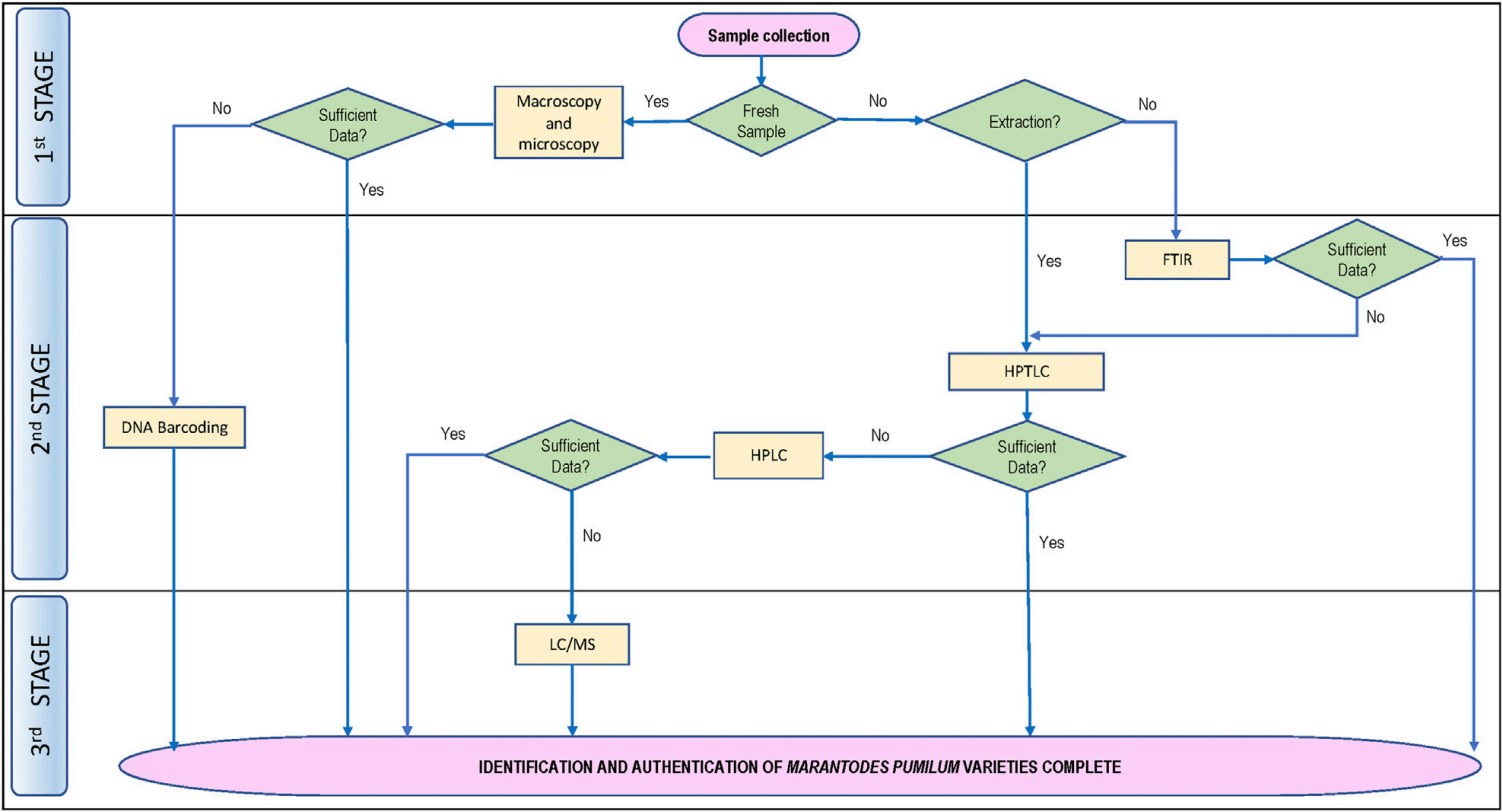
Proposed flow chart for authentication of *Marantodes pumilum* varieties.

The various techniques used to identify *M. pumilum* varieties and plant parts in the articles reviewed in this study (Table 1, 2), such as macroscopy, microscopy, chromatography, spectroscopy and chemometrics, suggest that a combination of approaches is required to authenticate botanical drug substances and products. However, the existing reports did not address phytochemical variation of a plant variety or plant part collected from various locations, differences between those collected from the wild sources and those collected from cultivated sources, as well as adulteration with other plant parts or varieties. When producing a standardized botanical drug product, obtaining botanical drug substance from a cultivated plantation location rather than the wild will assure plant homogeneity. Phytochemical indicators are frequently used to standardize botanical drug products. A guideline for selecting marker substances for quality control of botanical drug is provided by the World Health Organization (2017). To facilitate correct identification and authentication of *M. pumilum* utilized in botanical drug products development, it is vital to identify the phytochemical constituents with known therapeutic activity. As shown in Figure 4, a flow procedure for *M. pumilum* authentication up to the variety level is proposed based on an existing approach.

## Conclusion

This review found that no one technique for authenticating *M. pumilum* botanical drug substances and products can be used. Each technique has its own distinct interpretation of the plant, ranging from simple morphological characteristics to a more comprehensive comprehension of the *M. pumilum*’s phytochemical constituents. Developing proper authentication procedures is critical for the development and manufacturing of botanical drugs, whether for clinical trials or before the product reaches the consumer. Thus, additional research is necessary to determine the most effective authentication techniques for differentiating the varieties of *M. pumilum* and their plant parts to ensure that the correct species is used in the manufacturing process of botanical drug products and to avoid adulterations that could pose a health risk to consumers.

## Data Availability

The raw data supporting the conclusions of this article will be made available by the authors, without undue reservation.
